# Breast metastasis 18 years after nephrectomy for renal cell carcinoma: a case report

**DOI:** 10.1093/jscr/rjac116

**Published:** 2022-04-30

**Authors:** Ihssan Elouarith, Yassine Bouhtouri, Salma Elmajoudi, Salma Bekarsabein, Soumaya Ech-charif, Mouna Khmou, Hamza Messaoudi, Youssef Mahdi, Hafid Hachi, Basma El khannoussi

**Affiliations:** 1 Pathology Department, Oncology National Institute, Faculty of Medicine and Pharmacy, Mohammed V University, Rabat, Morocco; 2 Department of Surgical Oncology, Oncology National Institute, Faculty of Medicine and Pharmacy, Mohammed V University, Rabat, Morocco

## Abstract

Metastasis of renal clear cell carcinoma (RCC) to the breast is exceptional. Breast metastases of extra-mammary tumors are rare and usually involve melanoma, lymphoma or leukemia. We report the case of a patient with breast metastasis of renal clear cell carcinoma occurring 18 years after nephrectomy. A history of RCC should always raise suspicion about breast metastasis, a situation that remains exceptional and whose diagnosis relies on anatomopathology.

## INTRODUCTION

Breast metastasis of extra-mammary tumors is rare and usually involves melanoma, lymphoma or leukemia [1]. Metastasis of renal clear cell carcinoma (RCC) to the breast is exceptional [2, 3]. We report the case of breast metastasis of RCC, occurring 18 years after nephrectomy.

## CASE PRESENTATION

The patient was 69 years old and had undergone surgery 18 years ago for a right renal tumor for which the diagnosis of clear cell renal cell carcinoma was confirmed on the anatomopathological study of the surgical excision specimen.

**Figure 1:**
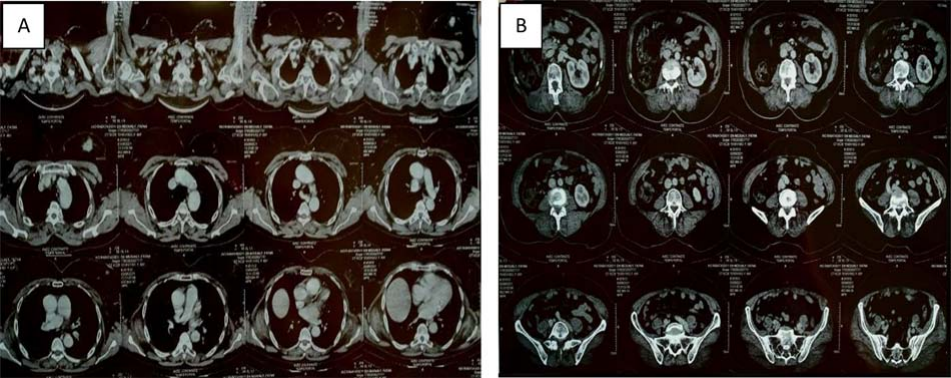
Thoracic-abdominal and pelvic computed tomography (CT) images showing on the thoracic level (**A**) a poorly limited mass measuring 27 × 22mm in the upper outer quadrant of the left breast (**B**) and a single left kidney at the abdomino-pelvic level.

Six months ago, the patient was referred to our facility by a general practitioner for the management of a nodule of the left breast, with a left breast mass in the upper external quadrant, tissue, about 2.5 cm long axis, poorly limited and irregular (Fig. 1).

**Figure 2:**
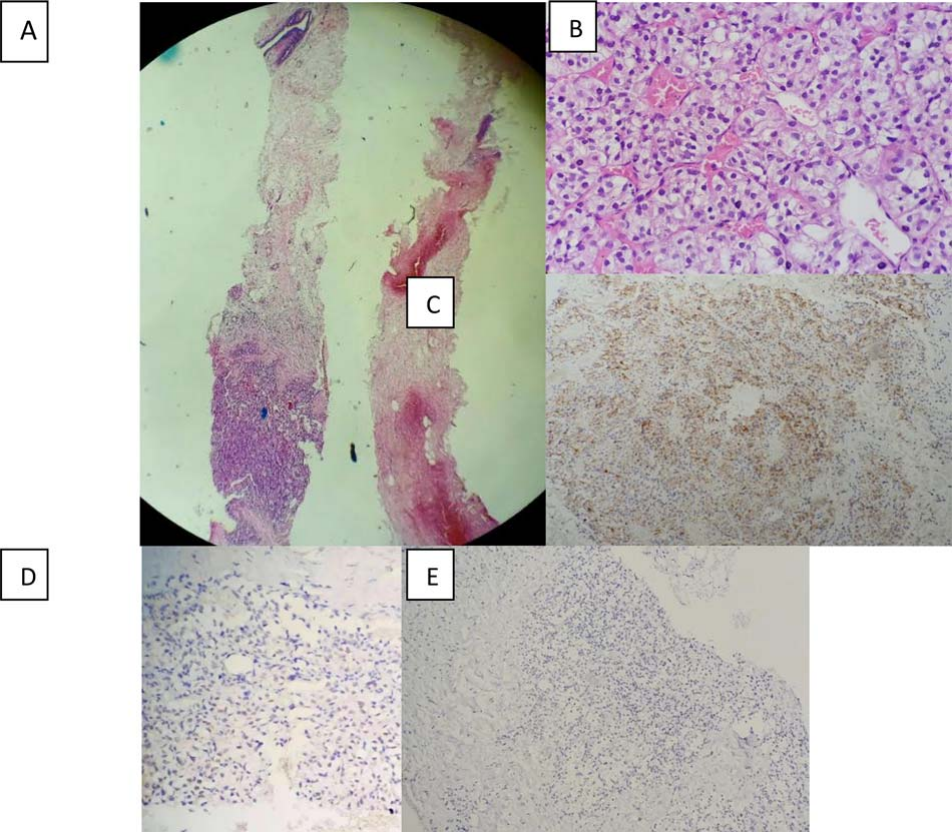
Morphological and immunohistochemical aspect: (**A**) Breast localisation of tumor proliferation (hematoxylin-eosin, magnification_40). (**B**)consisting of nests of cells with clear cytoplasm and a regular network of blood vessels (hematoxylin-eosin, magnification_400). (**C**) Immunohistochemical staining showing positive expression of CD10 in tumor cells (magnification_100). (**D**) Positive nuclear expression of PAX8 (magnification_200). (**E**) and negative expression of GATA3 (magnification _100).

The patient underwent a biopsy of the mass. The anatomopathological examination included a carcinomatous tumor proliferation of alveolar architecture composed of cells with clear cytoplasm surrounded by distinct cell membranes. Nests of tumors are separated by the delicate vascular network. Immunohistochemical staining showed that the tumor cells were positive for paired box gene (PAX) 8 and cluster designation or cluster of differentiation (CD) 10 but negative for GATA binding protein 3 (GATA3) and CK7 (Fig. 2). Faced with this morphological and immunohistochemical aspect, the diagnosis of breast localization of a clear cell renal carcinoma was made.

The patient underwent a lumpectomy whose anatomopathological study confirmed the diagnosis of breast metastasis of RCC.

## DISCUSSION

RCC accounts for 3% of all adult malignancies [4]. RCC metastasizes often to the lung, bone, lymph nodes, liver, adrenal gland and brain [5]. Metastasis to the breast remains exceptional [2, 3]. Breast metastases from extra-mammary malignancies are a rare situation and concern mainly lymphoma, leukemia, melanoma as well as ovary, stomach and lung [1]. Metastatic breast tumors may present as a single or multiple lesions. Radiographically, they are usually well-circumscribed without calcifications, whereas primary breast tumors often show speculations and/or microcalcifications [6, 7].

Anatomopathological study plays an indispensable role in the diagnosis of certainty of a breast metastasis of clear cell renal cell carcinoma. A morphological study under the microscope shows nests and sheets of tumor cells that are typically separated with a regular network of small and thin-walled blood vessels. Uncommonly, another minor pattern including tubular, papillary and trabeculae may be focally present. Tumor cells have well-defined cell membranes with clear cytoplasm that contain abundant cytoplasmic lipid and glycogen. Nucleoli may be small or conspicuous and prominent depending on the grade of the tumor. In high grade tumors, cells show a high nuclear grade and may present granular eosinophilic cytoplasm [8]. The immunohistochemical study allows conforming the renal origin of the tumor in front of positive nuclear staining of the tumor cells by anti-PAX8 antibodies and positive staining by CD10, anti-cytokeratin and anti-vimentin antibodies compatible with clear cell renal carcinoma. It also differentiates lesions from primary breast tumor proliferation by negative immunostaining with anti estrogen receptor (RE), progesterone receptor (RP), human epidermal growth factor receptor-2 (Her2)neu and GATA3 antibodies. Other breast metastases from extra-mammary malignancies present a problem of differential diagnoses, such as melanoma which can be eliminated after negative immunostaining for melanocyte markers human melanoma black (HMB-45) and adrenal cortical carcinoma characterized by positive staining of tumor cells by inhibin, Melana and steroidogenic factor 1 (SF1) but negative for PAX8, Epithelial membrane antigen (EMA) and keratins [9].

A history of RCC should always raise suspicion of breast metastasis; RCC may recur after nephrectomy with an interval that varies from a few months to several years. The route of RCC metastasis to the breast is considered to be hematogenous [10].

In general, breast metastases of any cancer have a poor prognosis with a mean survival of 10 months [11]. For patients with RCC metastases, the 5-year survival rate after ablation was 53%, compared with 5% in cases where ablation was not performed [12]. In the face of this finding, resection should always be considered in cases of solitary RCC metastasis to improve the prognosis.

## CONCLUSION

A history of RCC should always raise suspicion for breast metastases, a situation that remains exceptional and whose diagnosis relies on anatomopathology.
